# La dialyse péritonéale chez les patients de moins de vingt ans: expérience d'un centre hospitalier universitaire marocain

**Published:** 2012-06-23

**Authors:** Intissar Haddiya, Hakima Rhou, Fatima Ezaitouni, Naima Ouzeddoun, Rabia Bayahia, Loubna Benamar

**Affiliations:** 1 Service de néphrologie-dialyse-transplantation rénale CHU Ibn Sina Rabat, Maroc

**Keywords:** Adolescent, dialyse péritonéale, enfant, épuration extra-rénale

## Abstract

**Introduction:**

La dialyse péritonéale (DP) est une méthode d'épuration extra-rénale qui offre plusieurs avantages chez l'enfant et l'adolescent. Le but de cette étude est de rapporter notre expérience de DP chez des patients jeunes âgés de moins de vingt ans, et soulever l'intérêt et les avantages de cette technique chez cette catégorie de patients.

**Méthodes:**

Il s'agit d'une étude rétrospective incluant tous les dialysés péritonéaux âgés de moins de vingt ans au début du traitement par DP. Les paramètres de DP ainsi que les données cliniques et biologiques ont été recueillit et analysés.

**Résultats:**

Parmi 41 dialysés péritonéaux dans notre centre, huit sont des enfants et adolescents. L'âge moyen de nos patients est 16,1±2,6. Le sexe ratio M/F est égal à 1,6. 37,5% de nos patients ont une activité professionnelle et 62,5% ont une activité scolaire. Ils ont tous gardé leur activité toute la durée du traitement par DP. A l'initiation de la DP, 62,5% étaient hypertendus alors qu'actuellement 25% seulement sont hypertendus et aucun patient ne présente de signes cliniques de surcharge. Le maintien de la fonction rénale et résiduelle (FRR) et une amélioration de l'anémie a été noté chez nos patients.

**Conclusion:**

Notre expérience de DP chez l'enfant et l'adolescent est globalement satisfaisante. La DP a procuré plusieurs avantages dont le maintien d'une FRR, l'équilibre hydro-électrolytique, ainsi qu'une vie sociale éducative pour l'enfant et un maximum d'indépendance chez l'adolescent. Cette technique doit donc être encouragée surtout chez cette catégorie de patients.

## Introduction

La dialyse péritonéale (DP) est une méthode d’épuration extra-rénale (EER) qui peut-être proposée en première intention dans le traitement de l'insuffisance rénale chronique (IRCT) [1]. Elle repose sur des échanges de solutés et de solvant à travers le péritoine par un cathéter implanté dans le cul de sac Douglas [2, 3]. C'est une dialyse douce, continue, qui préserve la fonction rénale résiduelle, et qui peut–être effectuée à domicile [4].

Dans notre pays, la DP a vu le jour dans les années quatre-vingt puis elle a été abandonnée. Ce n'est qu'en Juillet 2006, qu'elle a été pour la première fois introduite parmi les différentes modalités d'EER au CHU Ibn Sina de Rabat. Cette technique a bénéficié ces dernières années d'avancées importantes qui ont permis d'une part d'en diminuer les complications, et d'autre part d'en optimiser l'efficacité [4]. Ses avantages sont évidents chez l'enfant et l'adolescent en attente d'une transplantation rénale (TR). En effet, la DP procure une vie relativement normale à l′enfant, et un traitement à domicile avec une implication familiale importante, sans entrave à l'activité scolaire. D'un autre côté les impératifs sociaux chez l'adolescent orientent souvent le choix vers la DP, qui permet grâce à sa flexibilité, de lui procurer une certaine indépendance.

Le but de cette étude est de rapporter une expérience satisfaisante de DP chez des enfants et adolescents suivis dans notre formation, de décrire leur profil clinico-biologique et évolutif, et soulever l'intérêt et les avantages de cette technique chez cette catégorie de patients.

## Méthodes

La DP a été adoptée dans notre centre en Juillet 2006. En Décembre 2009, nous avons réalisé une étude rétrospective incluant tous les dialysés péritonéaux âgés de moins de vingt ans au début du traitement par DP. Les paramètres étudiés sont d'ordre:

### 

#### Démographique

nous avons déterminé le sexe et l’âge au moment de la prise en charge en DP.

#### Social

nous avons défini le niveau scolaire comme suit: (Bon pour le niveau universitaire, moyen pour le niveau secondaire, bas pour le niveau primaire), l'activité professionnelle et scolaire, l'existence ou non de couverture médicale, les caractéristiques du logement (possibilité de stockage du matériel de DP et réalisation des échanges).

#### Clinique

nous avons noté la néphropathie causale, la présence ou non d'hypertension artérielle (HTA) définie selon l'organisation mondiale de la santé (OMS) par des chiffres supérieur ou égal à 140/90 mmHg, L'existence ou non d'oedème des membres inférieurs (OMI), la diurèse de 24 heures.

#### Biologique

nous avons relevé le taux d'hémoglobine (Hb), le taux de ferritine avec une valeur normale de 200 à 500 ng/ml, le taux de protides, la glycémie, la clairance rénale résiduelle calculée par la formule UV/P de la créatinine et de l'urée. Nous avons évalué la qualité des échanges péritonéaux et l'adéquation par l'appréciation du KT/V dialytique de l'urée et l’épuration des marqueurs suivants: le potassium, le phosphore, la réserve alcaline et l'acide urique.

A noter que les différents paramètres clinico-biologiques ont été évalués avant la mise en route de la technique de DP, 1 mois après et à la fin de l’étude.

Pour l'insertion du cathéter péritonéal, nous avons précisé le délai en jours entre la mise en place du cathéter et le 1er échange. La modalité d’échange utilisée est la dialyse péritonéale continue ambulatoire (DPCA). Nous avons précisé le nombre d’échanges par jour, sachant qu'un échange comporte trois étapes successives: L'infusion (introduction du dialysat préalablement réchauffé à 37°, dure 10 à 20 minutes); la diffusion ou stase ( 4 à 6 heures le jour et de 10 à 12 heures la nuit); le drainage (sortie du liquide après la stase, dure 10 à 20 minutes).

Pour l’évaluation de la qualité du péritoine, nous avons pratiqué le test d’équilibration péritonéale PET (peritoneal equilibration test) après minimum 6 semaines du début d’échange. Ainsi, on définit quatre catégories de perméabilité péritonéale: Hypoperméabilité péritonéale franche, hypoperméabilité péritonéale modérée, hyperperméabilité péritonéale modérée et hyperperméabilité péritonéale franche.

Nous avons aussi relevé les différentes complications en rapport avec la technique au cours de l’évolution. Ces complications sont de 2 types:

Les complications liées au cathéter péritonéal qui se divisent en 2 groupes: Les complications non infectieuses (mécaniques) liées soit au dysfonctionnement du cathéter péritonéal à savoir la migration, l'aspiration épiploïque ou l'obstruction du cathéter par la fibrine ou par un caillot de sang, soit à l'augmentation de la pression intra-péritonéale à savoir la fuite pleuro-péritonéale et génitale ou la hernie inguinale et ombilicale. Les complications infectieuses représentées par la tunnelite et la péritonite. Les complications au niveau du site d’émergence du cathéter à savoir le bourgeon, l'infection, l’écoulement et la fuite. L'analyse statistique a été faite par le logiciel SPSS version 10.0 for Windows.

## Résultats

### Paramètres démographiques

Parmi 41 patients dialysés péritonéaux dans notre centre, huit sont des enfants et adolescents ayant une croissance normale selon les courbes standards. L’âge moyen de nos patients est 16,1±2,6 ans avec des extrêmes allant de 10 à 18 ans. Le sexe ratio M/F est égal à 1,6. La néphropathie initiale est une glomérulonéphrite chronique dans quatre cas (50%). Il s'agit d'une néphropathie indéterminée dans un cas (12,5%) et une néphropathie de reflux dans deux cas (25%). Tous nos patients sont socialement actifs: Trois patients (37,5%) ont une activité professionnelle et les cinq autres (62,5%) ont une activité scolaire (Tableau 1).

**Tableau 1:** Paramètres démographiques de nos patients dialyses péritonéaux

Patient	Age	Sexe	Néphropathie initiale	Niveau scolaire	Activité professionnelle
1	18 ans	Masculin	indéterminée	Bon (lycéen)	non
2	17 ans	Féminin	Néphropathie de reflux	Bon (lycéenne)	non
3	10 ans	Féminin	Glomérulonéphrite chronique	Bon (CM2)	non
4	18 ans	Féminin	Glomérulonéphrite chronique	Moyen	oui
5	18 ans	Masculin	Glomérulonéphrite chronique	Moyen	oui
6	17 ans	Masculin	Glomérulonéphrite chronique	Bon	non
7	16 ans	Masculin	Néphronophtise	Moyen	oui
8	15 ans	Masculin	Néphropathie de reflux	Bon	non

### Modalités de DP

La mise en place des cathéters de DP est réalisée chirurgicalement sous sédation dans tous les cas. Le délai médian entre la pose du cathéter et le premier échange est de 4,6 jours (0-9) jours. La durée moyenne en DP est de 18,18±10,6 mois (5à 35) mois (Tableau 2).

**Tableau 2:** Modalités de dialyse péritonéale chez nos patients dialysés

Patient	Délai pose cathéter DP/ échange (en jours)	Durée DP (mois)
1	9 jours	19 mois
2	3 jours	35 mois
3	0 jours	5,5 mois
4	9 jours	26 mois
5	2 jours	21 mois
6	2 jours	24 mois
7	4 jours	5 mois
8	8 jours	10 mois

DP: Dialyse péritonéale

### Modalités d’échanges

Le nombre des échanges au début du traitement est répartit comme suit: 3 échanges ventre vide la nuit 6j/7 dans 25% des cas. 4 échanges ventre plein 7j/7 dans 50% des cas. 4 échanges ventre plein 6j/7 dans 25% des cas.

#### Clinique

A l'initiation de la DP, cinq patients (62,5%) étaient hypertendus, quatre patients (50%) avaient des oedèmes des membres inférieurs (OMI) alors qu'actuellement deux patients (25%) seulement sont hypertendus et aucun patient ne présente d'OMI. La diurèse résiduelle moyenne était de 1240±850 ml au début de la DP, actuellement elle est à 700±430 ml (Tableau 3).

**Tableau 3:** Evolution des paramètres cliniques chez nos patients dialysés péritonéaux

Paramètres	Avant DP (n: 8)	1mois (n: 8)	Actuellement (n: 7)
Hypertension artérielle	5 cas	5 cas	2 cas
Œdème des membres inférieurs	4 cas	3 cas	0 cas
Diurèse moyenne (ml)	1240±850	1130±600	700±430

n: nombre de patients; DP: dialyse péritonéale.

#### Biologie

Chez nos jeunes patients dialysés péritonéaux, la fonction rénale résiduelle (FRR) moyenne avant la DP était de 3,97±2,13 ml/min, elle est restée stable tout au long de la période de l’étude puisqu'actuellement elle est de 3,8±2,18 ml/min. Une amélioration de l'anémie est également notée avec un taux d'hémoglobine moyenne à 5,6±2,2 g/dl avant la DP et 9,6±1,7 g/dl à la fin de l’étude. Le fer injectable a été administré chez 12,5% de nos patients, les agents stimulants d’érythropoïétine dans 37,5% des cas et aucun de nos patients n'a été transfusé. Aucun cas de dénutrition n'a été observé, vu que la protidémie moyenne a demeuré inchangée pendant l’étude avec des chiffres de l'ordre de 58±3,11 mg/l avant la DP, et 59±3,13 mg/l actuellement. Aussi n'avons –nous pas noté d'hyperglycémie chez nos patients traités par DP, ceci est clairement démontré par les glycémies moyennes, 0,8±0,25 g/l et 0,9±0,2 g/l respectivement avant la DP et à la fin de l’étude (Tableau 4).

**Tableau 4:** Evolutions des paramètres biologiques chez nos patients dialysés péritonéaux

Paramètres	Avant DP (n: 8)	1 mois après DP (n: 8)	Actuellement (n:7)
Clairance rénale résiduelle moyenne (ml/min)	3,97±2,13	3,43±1,8	3,8±2,18
Hémoglobine moyenne (g/dl)	5,6±2,2	7,7±2,1	9,6±1,7
Protidémie moyenne (mg)	58±3,11	62±2,2	59±3,13
Glycémie moyenne (g)	0,8±0,25	1,1±0,12	0,9±0,2

n: nombre de patients; DP: dialyse péritonéale.

#### Qualité de l’épuration

La DP a permis de suppléer la fonction épurative rénale avec une baisse de kaliémie moyenne de 5,05±0,6 mmol/l avant DP à 4,7±0,45 mmol/l à la fin de l’étude. Les phosphorémies et uricémies moyennes ont respectivement baissé de 63,8±5,16 mg/l et 67,2±4,21 mg/l au début de l’étude à 52±4,2 mg/l et 65,5±4,15mg/l actuellement (Tableau 5).

**Tableau 5:** Qualité d’épuration chez nos patients dialysés péritonéaux

Nombre de patients (n)	Avant DP (n: 8)	1 mois après DP (n: 8)	Actuellement (n:7)
Kaliémie moyenne (mmol/l)	5,05±0,6	4,1±0,25	4,7±0,45
Phosphorémie moyenne (mg/l)	63,8±5,16	45,65±3,25	52±4,2
Uricémie moyenne (mg/l)	67,25±4,21	59±5,13	65,5±4,15
KT/V dialytique de l'urée			2,1±1,5

n: nombre de patients; DP: dialyse péritonéale

### Evolution et complications

#### Non infectieuses

Nous avons noté une migration du KT dans deux cas (25%).

#### Infectieuses

Nous avons observé un épisode de péritonite dans trois cas (37,5%) avec une bonne évolution sous traitement antibiotique. L'infection du site d’émergence a également été notée dans trois cas (37,5%) avec bonne évolution sous traitement antibiotique local et général.

### Transplantation rénale

Une patiente de 18 ans a été transplantée par le rein de sa mère, avec bonne évolution actuellement à un mois post-greffe et reprise rapide de la fonction rénale. Tous nos autres patients sont également candidats à la TR, mais 50% d'entre eux n'ont pas de donneur vivant apparenté (DVA). Un seul patient a un DVA mais refuse la TR, et désire rester en DP.

## Discussion

La DP est une méthode d'EER qui peut être proposée en première intention dans l'IRCT. Elle est basée sur les échanges des solutés et du solvant à travers le péritoine, selon un gradient de concentration, de pression osmotique et hydrostatique [1–3]. Les progrès du matériel actuellement disponible ont permis d'en réduire les complications infectieuses, d'assurer une meilleure tolérance et une protection prolongée de la membrane péritonéale [4, 5]. Cette technique présente de multiples avantages chez l'enfant et l'adolescent. En effet, elle procure non seulement une vie relativement normale à l′enfant, mais aussi lui permet-elle une croissance normale (comme c'est le cas de nos patients) et un traitement à domicile avec une implication familiale importante [2]. D'un autre côté les impératifs sociaux chez l'adolescent orientent souvent le choix vers la DP, qui permet grâce à sa flexibilité, de lui procurer une certaine indépendance [2, 3].

Des études ont montré que les indices de qualité de vie et le taux d'emploi sont supérieurs chez les personnes en DP [6, 7]. Dans notre série, tous nos patients étaient actifs au début de la DP (37,5% professionnellement actifs et 62,5% étudiants) et le sont restés tout au long du suivi en DP, ce qui rejoint les données de la littérature. De plus, La DP en première intention permet un meilleur contrôle de la pression artérielle et de l’état hydrique pendant les 1ères années de dialyse [8]. Dans notre série, l'hypertension artérielle a été retrouvée initialement chez 62,5% de nos patients, et à la fin de l’étude, seuls 25% de nos patients étaient toujours hypertendus, et aucun cas ne présentait un oedème des membres inférieurs. Aussi, la dénutrition est une complication fréquente en DP [5]. Dans notre étude, aucun de nos patients n'a développé d'hypoprotidémie. En DP, on obtient une normalisation des taux d'hémoglobine plus élevés sous des doses faibles d’érythropoïétine. Dans une étude rétrospective portant sur 121,970 patients en HD et 7129 en DP, on a pu montrer que les besoins en érythropoïétine sont moindres en DP qu'en HD [9]. Dans notre étude, nous avons observé que les besoins en érythropoïétine ne sont pas très importants et nous n'avons eu recours à la transfusion sanguine chez aucun de nos patients.

La DP chez le sujet jeune en attente de TR est une excellente indication [10]. La survie du greffon est identique chez les patients préalablement en DP ou en HD [10, 11]. Une amélioration de la fonction rénale du greffon est plus rapidement obtenue chez les patients antérieurement en DP [11], et c′est le cas pour notre jeune patiente transplantée, qui après une semaine de TR a normalisé ses chiffres de créatinine. Pour le reste de nos patients candidats à la TR et qui sont au nombre de cinq, 50% d'entre eux n'ont pas de DVA. Un patient a un DVA mais refuse la TR et désire rester en DP. Ainsi, les avantages majeurs de la DP résident, en plus du maintien d'une FRR et la préservation du capital vasculaire, en une compliance mécanique intrapéritonéale propice à recevoir un greffon adulte lors de la TR [7].

## Conclusion

La DP chez l'enfant et l'adolescent permet une attente de TR en économisant le capital vasculaire, favorisant la persistance de la diurèse résiduelle et autorisant une vie sociale éducative pour l'enfant et un maximum d'indépendance chez l'adolescent. En effet, La technique de DP nouvellement introduite au Maroc, doit être encouragée et soutenue; d'autant plus qu'il existe une inégalité de répartition des centres d'hémodialyse au Maroc et une incapacité de prendre en charge tous les patients en IRCT. De plus, la TR n'est pas toujours possible dans notre pays.
